# A Multi-Response Investigation of Abrasive Waterjet Machining Parameters on the Surface Integrity of Twinning-Induced Plasticity (TWIP) Steel

**DOI:** 10.3390/ma18143404

**Published:** 2025-07-21

**Authors:** Onur Cavusoglu

**Affiliations:** Gazi University, Faculty of Technology, Department of Manufacturing Engineering, 06560 Ankara, Türkiye; onurcavusoglu@gazi.edu.tr

**Keywords:** abrasive waterjet, TWIP, surface roughness, kerf, taper angle, material removal rate

## Abstract

Twinning-induced plasticity (TWIP) steels represent a significant development in automotive steel production, characterized by advanced strength and ductility properties. The present study empirically investigated the effects of process parameters on the cutting process and surface quality of TWIP980 steel sheet by abrasive water jet (AWJ) cutting. The cutting experiments were conducted on 1.4 mm thick sheet metal using four different traverse speeds (50, 100, 200, and 400 mm/min) and four different water jet pressures (1500, 2000, 2500, and 3000 bar). Two different abrasive flow rates (300 and 600 g/min) were also utilized. The cut surfaces were characterized in three dimensions with an optical profilometer. The parameters of surface roughness, kerf width, taper angle, and material removal rate (MRR) were determined. Furthermore, microhardness measurements were conducted on the cut surfaces. The optimal surface quality and geometrical accuracy were achieved by applying a combination of parameters, including 3000 bar of pressure, a traverse rate of 400 mm/min, and an abrasive flow rate of 600 g/min. Concurrently, an effective cutting performance with increased MRR and reduced taper angles was achieved under these conditions. The observed increase in microhardness with increasing pressure is attributable to a hardening effect resulting from local plastic deformation.

## 1. Introduction

The primary goals in many areas, particularly in the automotive sector, are to reduce vehicle weight, minimize carbon footprint, and improve passenger safety [1,2,3,4]. In the context of material development studies, improved high strength steels have recently been implemented. Twinning-induced plasticity (TWIP) steels, which are classified as second-generation improved high-strength steels, exhibit a remarkable capacity for absorbing high energy levels during deformation. This property is attributed to their high elongation capability and substantial strength values [5,6,7,8,9]. Various cutting methods can be used to remove sheet material, including laser cutting, abrasive waterjet cutting, and die cutting [10]. The evaluation of these methodologies can result in alterations to the mechanical properties of sheet metal due to elevated levels of heat generated during laser cutting processes [11]. The die-cutting process requires the fabrication of a separate die for each geotherm to be cut, resulting in increased cost and time [12]. In the abrasive water jet cutting process, abrasive sand grains are directed onto the material’s surface to be cut under high pressure, thereby resulting in the material’s removal. A notable benefit of this process is the absence of heat input [13]. Furthermore, the capacity to process a variety of thicknesses and materials during the cutting process and the use of minimal processing forces can be seen as additional advantages of abrasive waterjet cutting [12,13,14]. In the abrasive waterjet cutting process, the processing parameters that significantly impact the machining process are, in addition to the material properties, the travel speed, the head, the grit ratio, and the nozzle height [15,16].

In the studies referenced in the literature, Kumar et al. [17] investigated the abrasive waterjet machining process of Inconel 718 material using the response surface method. The parameters of greatest influence have been determined to traverse speed and abrasive flow rate [17]. Llanto et al. [18] investigated the effects of traverse speed and material thickness on the machining process in abrasive waterjet machining of 304 L grade austenitic stainless steel. As traverse speed increases, the material removal rate and kerf taper angle concomitantly rise [18]. Dumbhare et al. [19] performed multi-objective optimization for surface roughness and kerf taper angle in the machining process for 6 mm thick mild steel sheet with abrasive water jet. It was determined that a high abrasive flow rate and a low traverse speed would be more suitable for achieving optimal surface roughness and kerf taper angle [19]. Veerappan and Ravichandran conducted a study to investigate the effects of process parameters on kerf angle in water jet machining of 10 mm thick Waspaloy super alloy. A subsequent decrease followed the initial increase in kerf angle with increasing head size. This phenomenon has been observed to increase proportionately to the increase in traverse speed. The magnitude of the MRR exhibited an upward trend in conjunction with an augmentation in both the head and abrasive flow rates. Conversely, a decline in traverse speed resulted in a negative correlation with the MRR [20]. In Kant and Dhami’s study, the parameters of the abrasive waterjet process were investigated in the machining of 13 mm thick EN31 material. It has been reported that surface roughness is more influenced by traverse speed than initial speed. Furthermore, the effects of abrasive flow rate may not be sufficiently considered [21]. Singh and Sukla investigated the kerf characteristics of Inconel 600 material in abrasive waterjet machining by response surface methodology. As the traverse speed increased, the wheel width decreased. Similarly, as the head decreased, the wheel width decreased. Finally, as the abrasive flow rate decreased, the wheel width decreased. As traverse speed increased, the kerf taper angle decreased. Conversely, the kerf taper angle increased as pressure and abrasive flow rate increased [22]. Ahmed et al. investigated the effects of traverse speed and water pressure on abrasive waterjet cutting of 7 mm thick 7075 aluminum alloy material at different stand-off distances. The optimal surface quality was achieved at the closest stand-off distance, under conditions of low head pressure and high traverse speed [23]. Goshert et al. [24] investigated the effects of laser cutting, milling, electro-erosion, and abrasive water jet machining on the mechanical properties of QP980 and DP600 steel sheets during deformation after cutting. Abrasive water jet machining was found to favorably affect the highest uniform and total elongation capability [24]. Sheng et al. [10] conducted a comparative analysis of the electro-erosion, laser cutting, die cutting, and abrasive water jet cutting of high-Mn TWIP steel with a sheet thickness of 1.45 mm. The highest surface roughness value was obtained in the AWJ cutting process [10]. Ramakrishnan’s research focused on investigating the effects of pressure, stand-off distance, and abrasive flow rate (AFR) on Ti-6Al-4V waterjet cutting. The decrease in pressure was found to be associated with an increase in surface roughness. Similarly, an increase in the AFR was found to be associated with an increase in surface roughness [25]. Abushanab et al. [26] investigated the surface characteristics of a 2 mm thick Ti6Al4V alloy after abrasive water jet machining using the Taguchi method. Increasing pressure resulted in a surface of enhanced smoothness, while higher traverse speeds resulted in a surface quality deterioration. While the increase in AFR has been shown to enhance machinability, using an excessively high AFR has been demonstrated to exert a detrimental effect on machining processes [26]. Perec’s research focused on machining 5 mm thick Ti6AlV5 with AWJ, exploring the impact of varying process parameters. A decrease in traverse speed resulted in an increased depth of cut. Conversely, an increase in the AFR ratio from 15% to 20% had a positive effect, but a larger increase in AFR resulted in a slight decrease in depth of cut [27]. A comprehensive review of the extant literature reveals that the traverse speed, pressure, and abrasive flow rate are the primary parameters influencing the quality of the cut product in the water jet cutting process. Furthermore, it has been observed that optimizing these parameters is contingent upon the sheet thickness and the type of material utilized to ensure optimal product quality. Despite the extensive research on sheet cutting utilizing abrasive waterjet (AWJ) technology, no targeted study has been found in the literature addressing TWIP980 steel sheets. This study seeks to fill this gap by conducting a comprehensive investigation of the effect of critical AWJ parameters on the machinability of TWIP980 steel sheet. In light of the growing industrial significance of TWIP980 steel, particularly in critical automotive structural components such as B-pillars, reinforcement plates, and door inner impact beams, it is imperative to develop a comprehensive understanding of its behavior under AWJ processing. This study demonstrated the potential value of this approach as a potential alternative to conventional thermal cutting methods, particularly in cases where parts are susceptible to thermal impact zone formation.

The objective of this study is to examine the individual and combined impacts of traverse speed, water pressure, and abrasive flow rate on the performance of abrasive waterjet (AWJ) machining of TWIP980 steel sheets. The present study is concerned with the evaluation of surface roughness, kerf geometry (kerf width and taper angle), material removal rate (MRR), and microhardness as critical indicators of surface integrity and machining quality. The investigation aims to determine the most effective process parameters for improving cutting precision, reducing surface degradation, and encouraging strain-induced hardening on the cut surfaces of TWIP980. This approach enables the determination of optimal machining conditions, thereby ensuring the highest attainable surface quality and enhanced processing efficiency.

## 2. Materials and Experimental Procedure

**Figure 1:**
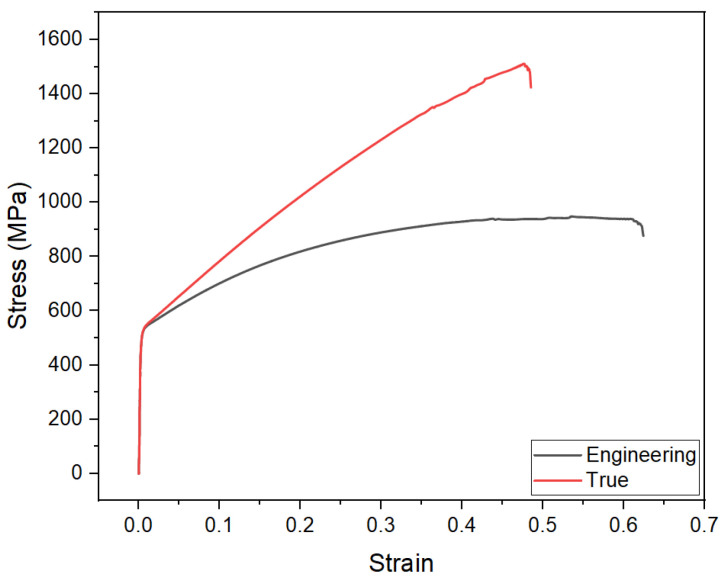
Engineering and true stress–strain curves of TWIP980 steel under uniaxial tensile loading.

In this study, cold rolled twinning-induced plasticity steel 980 (TWIP980) steel with a sheet thickness of 1.4 mm was utilized. The material was produced and supplied by POSCO Steel Co. (The Pohang Iron and Steel Company, located in Pohang, Republic of Korea). POSCO Steel Co. is a prominent industrial entity in the global steel industry. The chemical composition of TWIP980 steel is presented in Table 1. Figure 1 presents the engineering and true stress–strain curves of TWIP980 steel. As seen in the curves, the material exhibits a yield strength of approximately 550 MPa, followed by a continuous increase in stress reaching up to around 1479 MPa. The total elongation is determined to be approximately 58%.

The present study investigates the effect of machining parameters on the process of waterjet cutting of twinning-induced plasticity 980 (TWIP980) steel sheet material. A series of experimental studies were conducted on a steel sheet of TWIP980, with a thickness of 1.4 mm. Cutting operations were executed under varying conditions, encompassing traverse speeds, pressures, and abrasive flow rates. The experimental design employed the parameters and factor levels delineated in Table 2. The traverse speeds for the cutting processes were determined to be 50, 100, 200, and 400 mm/min. Water jet pressure levels range from 1500 to 3000 bar. Abrasive flow rates of 300 and 600 g/min were applied.

**Figure 2:**
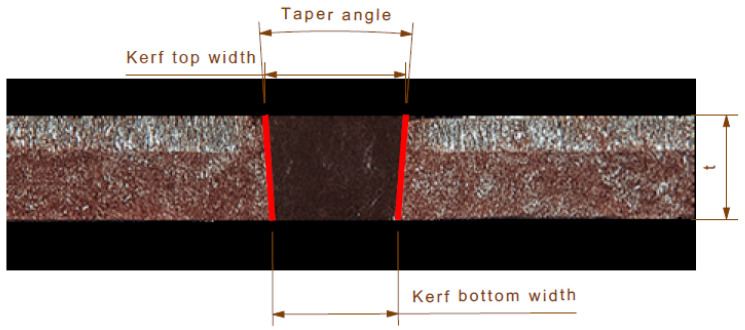
Kerf geometry of the cut sheet metal showing the top width, bottom width, and taper angle.

Following the cutting tests, the kerf width, taper angle, and hardness values of the waterjet processing parameters were measured on the cut specimens, and the surface topographies were analyzed. The material removal rate was then calculated. The kerf width and taper angle measurements were determined by measuring the pressure water inlet and outlet zones with the help of a optical microscope (Leica Microsystems GmbH, Wetzlar, Germany). The hardness values of the cut surfaces were measured using a Micro Vickers Hardness Tester (EMCO-TEST DuraScan 70 G5, EMCO-TEST Prüfmaschinen, GmbH, Salzburg, Austria). The measurements were obtained at 300 µm intervals, parallel to the direction of pressurized water flow. Four measurements were recorded for each sample, and the mean values were calculated for the analysis. The Vickers method was employed to measure the hardness, and a force of 1 kg (kgf) was applied. Subsequently, the surface topography of the cut surfaces was obtained with an optical profilometer (Phase View, Toulouse, France). Subsequently, material removals are performed to evaluate the effects of water jet parameters on the machining quality of the material. As illustrated in Figure 2, the kerf geometry is evident on the cut sheet [18,28].

The kerf taper angle was determined by calculating the measurements according to the relation given in Equation (1). In this regard, “W_t_” denotes the top kerf width, while “W_b_” denotes the bottom kerf width. The taper angle is denoted by “θ”, denoted by t, is defined as the thickness of the sheet. The material removal rate is calculated according to Equation (2). The material removal rate (MRR) is expressed as the product of sheet thickness (t), kerf width (W), and traverse speed (V), which is calculated as the ratio of length of cut (mm) to time of cut (min) [18,19].

(1) $$Kerf\;Taper\;Angle\;\theta =Arctan\;\frac{W_{t}-W_{b}}{2t}$$

(2) $$MRR=t\cdot \frac{W_{t}+W_{b}}{2}\cdot V$$

## 3. Results and Discussion

**Figure 3:**
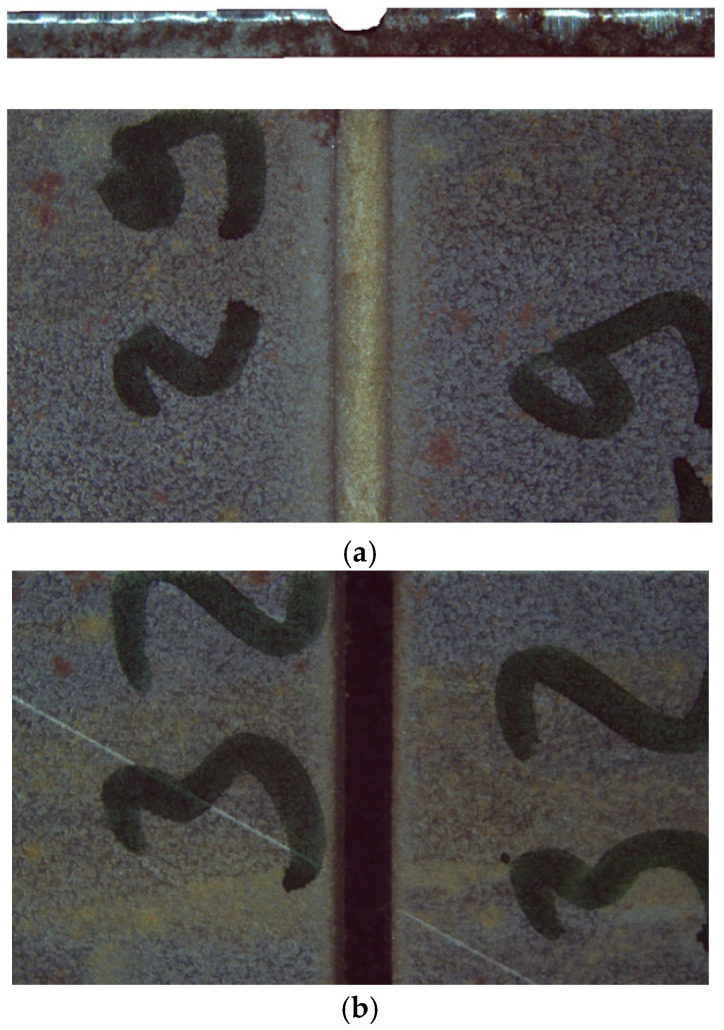
(**a**) An unsuccessful cutting experiment was performed at 1500 bar, 100 mm/min traverse speed, and 300 g/min abrasive flow rate (AFR). (**b**) A successfully cut specimen with a smooth and continuous kerf surface.

An evaluation of the experimental results obtained under varying parameters of abrasive flow rate, water jet pressure, and traverse speed is conducted. A comprehensive analysis examined the variations in kerf width, taper angle, hardness, and surface roughness characteristics. The findings of this study elucidated the intricate interplay of water jet cutting parameters on machining performance and cutting quality. Furthermore, the study delved into the determination of optimal cutting conditions for 1.4 mm thick TWIP steel sheet material. However, no cutting was observed at pressures of 1500 bar, with a traverse speed of 400 mm/min and an AFR of 300 g/min. In the case of these machining conditions, it was impossible to achieve adequate material removal from the sheet. Figure 3a presents the uncut surface image. As illustrated in Figure 3b, the image of the successfully cut sheet is presented. In all other machining conditions, cutting operations were performed successfully.

**Figure 4:**
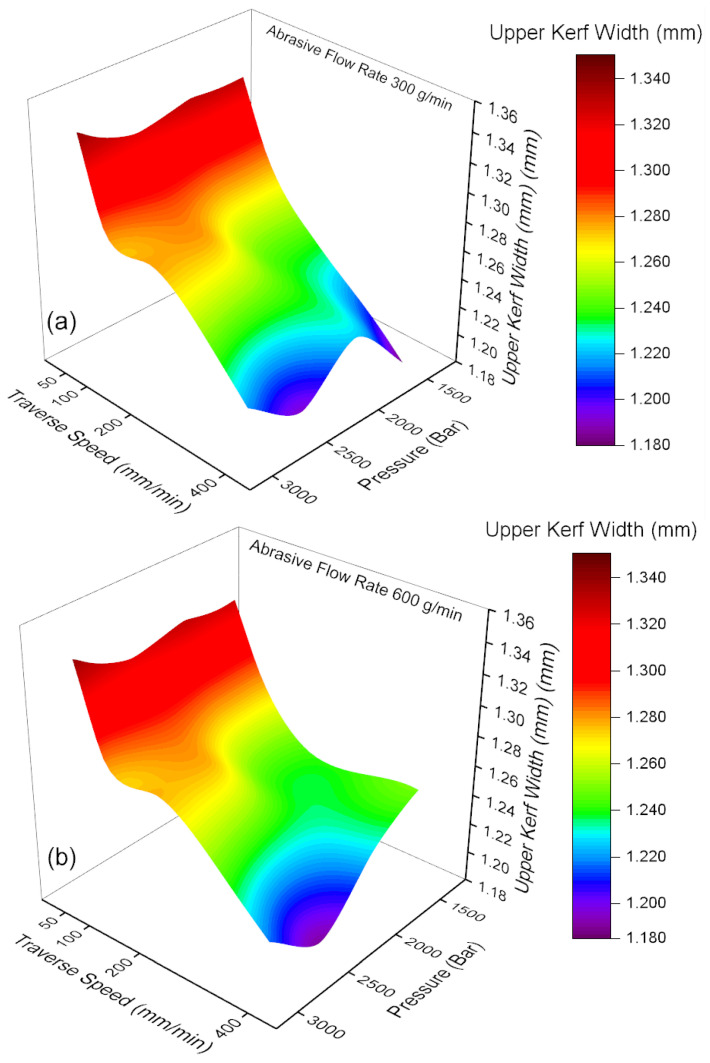
Traverse speed, pressure and upper kerf width relation: (**a**) 300 g/min abrasive flow rate; (**b**) 600 g/min abrasive flow rate.

The variation in kerf width, traverse speed, and pressure parameters depending on the abrasive flow rate are illustrated in Figure 4a,b. When Figure 4a is subjected to analysis, it becomes evident that the kerf width values exhibit a substantial variation in accordance with the traverse speed and pressure parameters, given a constant abrasive flow rate of 300 g/min. It has been demonstrated that the kerf width increases proportionately to the traverse speed and the pressure [29,30]. At low traverse speeds (50 mm/min) and low pressure levels (1500 bar), the kerf width reaches maximum values and hovers around 1.34 mm. This phenomenon is believed to be associated with the diminished focusing capability of the water jet under conditions of low pressure and speed, as well as greater variability in the impact of the abrasive particles on the material surface, resulting in irregular wear formation in the edge regions. The minimum kerf width value was determined to be approximately 1.18 mm at high pressure (3000 bar) and high traverse speed (400 mm/min). As demonstrated in Figure 4b, an increase in the abrasive flow rate to 600 g/min resulted in a decline in kerf width values, with most measurements falling to lower levels. As the traverse speed and pressure increase, the kinetic energy of the water jet and its capacity to focus on the cutting zone increases so that the kerf width may decrease. This finding aligns with the results of the study conducted by Dumbhare et al. [19]. An increase in the abrasive flow rate results in an increase in the number of particles carried into the water jet and the kinetic energy transmitted to the material surface. This, in turn, allows for more controlled and homogeneous cutting processes. However, under conditions of low traverse speeds and low pressure, kerf width values can persist at elevated levels. This phenomenon can be attributed to the fact that, despite the high abrasive flow rate, the water jet is unable to be adequately focused under low speed and pressure conditions [31]. The hypothesis of this study is that the process under investigation will result in surface wear effects on the material that are not consistent with the desired product specifications. A comparison of Figure 4a,b reveals that increasing the abrasive flow rate has a positive effect on kerf width. A combination of high pressure and high traverse speed results in reduced kerf widths at a flow rate of 600 g/min. This finding indicates that the cutting process exhibits increased homogeneity.

**Figure 5:**
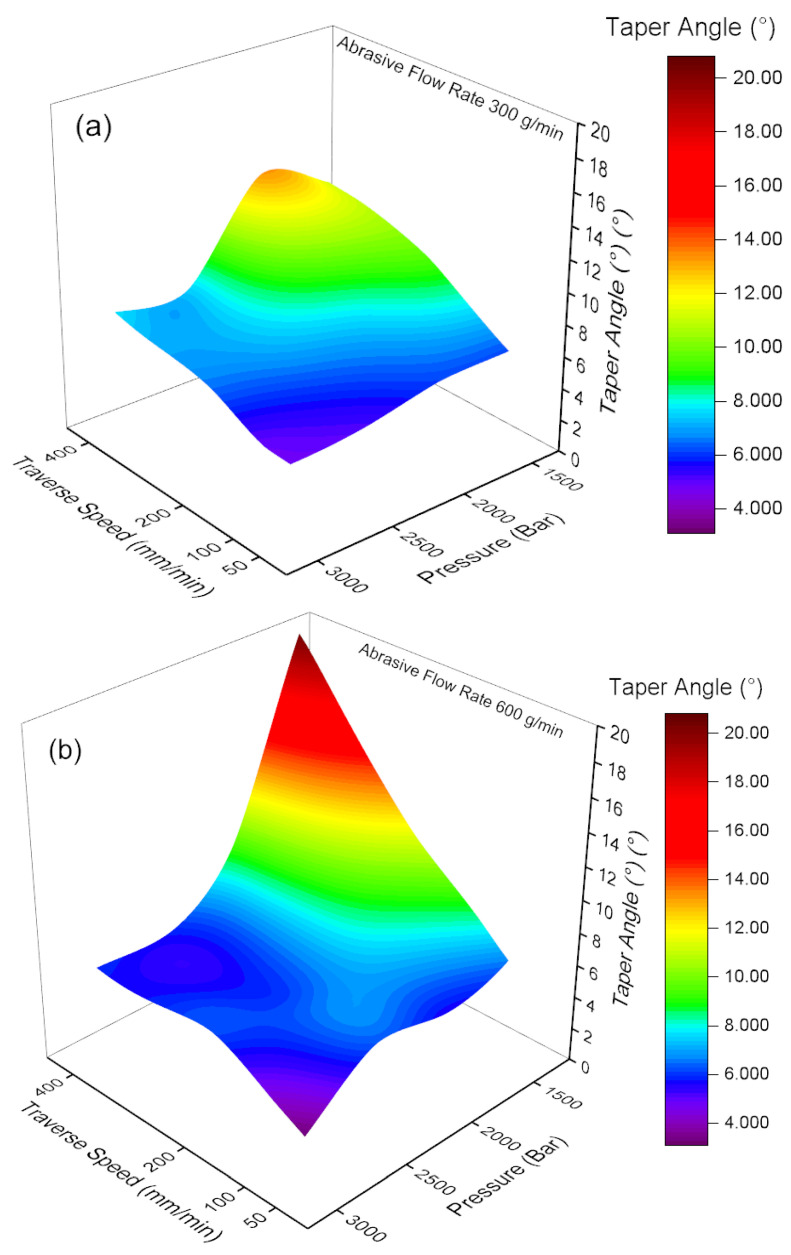
Traverse speed, pressure, and taper angle width relation (**a**) 300 g/min abrasive flow rate (**b**) 600 g/min abrasive flow rate.

The variations in taper angle, traverse speed, and pressure parameters depending on the abrasive flow rate ratio are given in Figure 5a,b. Upon examination of Figure 5a, it is determined that the taper angle values reach maximum levels at low traverse speeds (50 mm/min) and low pressure levels (1500 bar), with a mean value of approximately 20°. This phenomenon is believed to result from the formation of irregular and wide-angle cut marks on the material surface under conditions of low pressure and traverse speed. As the pressure and traverse speed increase, a significant decreasing trend is observed in the taper angle values. In conditions of high pressure (3000 bar) and accelerated travel speed (400 mm/min), the taper angle shows a reduction to a minimum of approximately 4.7°. The result indicates that the water jet exerts a more concentrated force on the surface of the sheet material, thereby producing a narrow and smooth cut. The present result is further corroborated by the findings of the study conducted by Llanto et al. [32]. It has been demonstrated that increasing the abrasive flow rate can result in a reduction in the taper angle [33]. As illustrated in Figure 5b, an increase in the abrasive flow rate to 600 g/min results in a general decrease in taper angle values, leading to lower levels. This phenomenon can be attributed to the increased abrasive flow rate, which leads to increased particles carried to the water jet and increased kinetic energy transmitted to the surface. Consequently, this results in a more homogeneous and controlled cutting process. A comparison of Figure 5a,b reveals that increasing the abrasive flow rate substantially enhances the taper angle. Specifically, when high pressure and high traverse speed are combined, lower taper angle values are obtained at an abrasive flow rate of 600 g/min. This indicates that the cutting process is narrower, smoother, and more controlled. In this case, the results support the hypothesis that an increase in the amount of abrasive material would positively contribute to the quality of the edge and geometric precision in the water jet cutting process.

**Figure 6:**
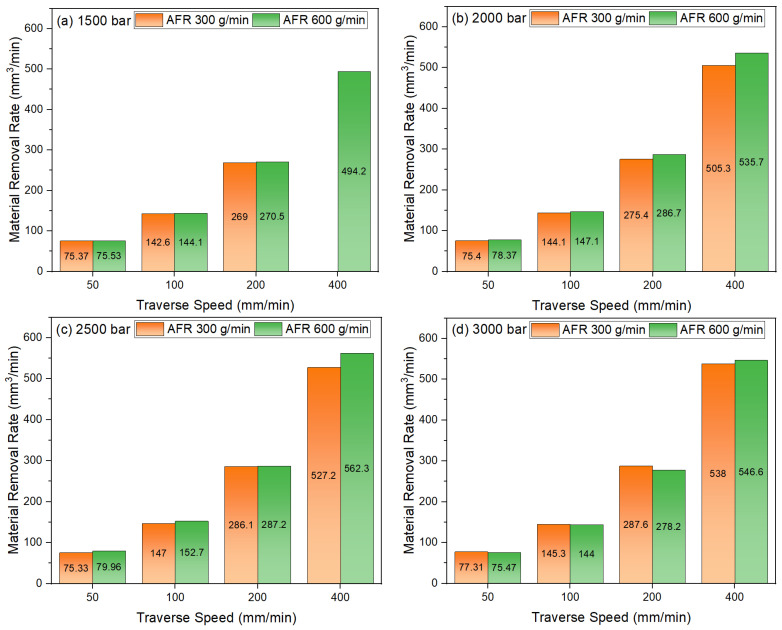
Traverse speed, pressure, and material removal rate relation: (**a**) 1500 bar; (**b**) 2000 bar; (**c**) 2500 bar; (**d**) 3000 bar.

The Material Removal Rate (MRR) results are presented in Figure 6 for various traverse speeds and pressure levels and for two distinct abrasive flow rates. An analysis of Figure 6a (1500 bar) reveals that the MRR values are at a low level (approximately 75–145 mm^3^/min) at low traverse speeds (50 and 100 mm/min). Furthermore, the effect of abrasive flow rate variation (300 g/min and 600 g/min) is limited at these speeds. To achieve an optimal material removal rate in the waterjet cutting of sheet metal, it is essential to use a combination of high pressure, high travel speed, and high abrasive flow rate [34]. As the traverse speed increases (200 and 400 mm/min), there is a significant increase in MRR values, up to approximately 494 mm^3^/min at 400 mm/min and 600 g/min flow rate. It is hypothesized that higher traverse speeds and abrasive flow rates will increase the material removal rate (MRR) by providing higher kinetic energy and greater abrasive particle density over a larger surface area. The results of Figure 6b (2000 bar) demonstrate that the general trend is analogous to that of 1500 bar, yet MRR values attain higher levels. At low traverse speeds, the MRR increase was found to be limited, while at high speeds (400 mm/min) with an abrasive flow rate of 600 g/min, an MRR of approximately 535 mm^3^/min was obtained. The higher hydrostatic pressure increased the kinetic energy of the water jet, thereby improving its cutting efficiency and accelerating the removal rate. As demonstrated in Figure 6c, which illustrates the impact of 2500 bar, it becomes evident that the effect of increasing pressure on the MRR is more pronounced. Furthermore, MRR values exhibit an increase at all traverse speeds. At higher traverse speed (400 mm/min), the maximum MRR was reached at approximately 562 mm^3^/min, accompanied by an abrasive flow rate of 600 g/min. This result shows that high pressure and high abrasive flow rate together maximize the material removal rate. As illustrated in Figure 6d (3000 bar), the effects of traverse speed and abrasive flow rate on MRR at the highest pressure level are demonstrated. At low speeds (50 and 100 mm/min), the material removal rate (MRR) values remained low; however, at high speeds (400 mm/min), MRR values of 538 and 547 mm^3^/min were obtained at flow rates of 300 and 600 g per minute, respectively. It has been observed that MRR values increase with increasing traverse speed and pressure. At all pressure levels, the effect of abrasive flow rate is limited at low traverse speeds, while this effect becomes more pronounced at high speeds. It has been demonstrated that there is a considerable increase in MRR values when the abrasive flow rate is augmented, particularly under circumstances involving elevated traverse speed and pressure.

**Figure 7:**
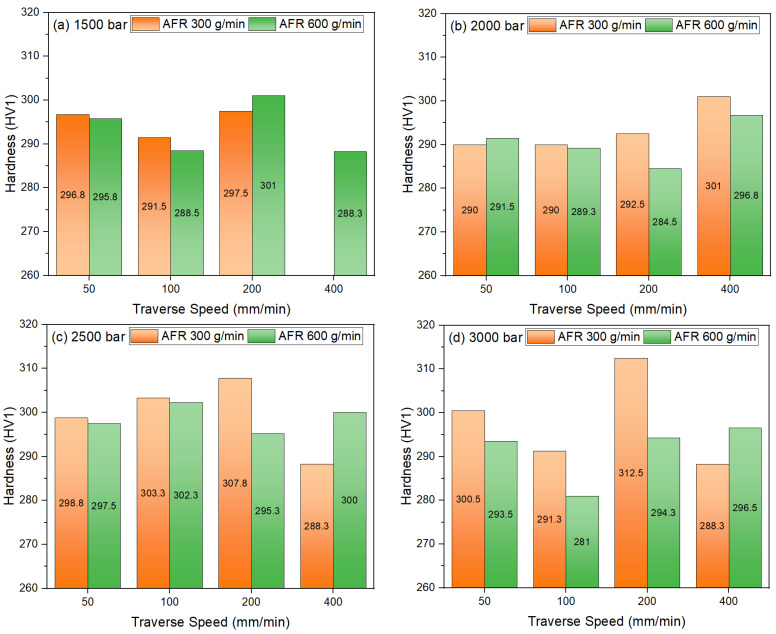
Traverse speed, pressure, and hardness relation: (**a**) 1500 bar; (**b**) 2000 bar; (**c**) 2500 bar; (**d**) 3000 bar.

Figure 7 illustrates the alterations in the microhardness (Vickers HV1) values of the surfaces obtained after the abrasive waterjet cutting of TWIP980 steel sheet material under varying processing conditions. The application of high water jet energy and high pressure has been demonstrated to result in increased surface deformation and hardness [35,36]. As shown in Figure 7a, the microhardness distribution of TWIP980 steel sheet material under 1500 bar pressure is evident. At an abrasive flow rate of 300 g/min, the measured values were 296.8 HV1 at 50 mm/min, 294.8 HV1 at 100 mm/min, and 297.5 HV1 at 200 mm/min. However, the cutting process could not be realized at a traverse speed of 400 mm per minute. This outcome indicates that the combined effect of low pressure and low abrasive ratio is unable to increase the specific energy of the abrasive waterjet sufficiently. At an AFR of 600 g/min, the value of 301.0 HV1 obtained at 200 mm/min is approximately 1.2% higher than at low AFR, suggesting that the increase in abrasive particle ratio provides more effective microstructure deformation. However, at a rate of 400 mm/min, the hardness reduces to 288.3 HV1, a phenomenon attributed to the diminished interaction time of the abrasive particles with the surface. While the deformation time is prolonged at low speeds, it is hypothesized that effective hardening is impeded due to the constrained abrasive density. Consequently, the optimal balance between AFR and traverse speed under low-pressure conditions is paramount. In Figure 7b, 301 HV1 value obtained for AFR 300 g/min under 2000 bar pressure is determined as the highest result at 400 mm/min traverse speed. This suggests that increasing pressure increases the flow rate in the abrasive waterjet. Thus, the impact energy of the abrasive particles increasing the amount of local strain in the cutting zone and thus increasing the dislocation density on the sheet metal cutting surfaces. The maximum hardness value of 289.3 HV1 was achieved at a traverse speed of 100 mm/min by applying an AFR of 600 g/min. At a speed of 200 mm/min, this value is reduced to 284.5 HV1. The increase in abrasive density has been shown to disrupt the coherence of the abrasive waterjet, potentially leading to irregular interactions between the abrasive particles. This phenomenon is believed to contribute to the irregularity of the deformation process and may have a deleterious effect on the resulting hardness. As Figure 7c shows, a value of 307.8 HV1 was recorded at a travel speed of 200 mm per minute, a flow rate of 300 g per minute, and an internal pressure of 2500 bar. This is the highest microhardness value that has been recorded at this pressure level. The higher pressure results in increased abrasive exit speeds and significant strain generation in the cutting zone, contributing to surface hardening by dislocation deposition. Under identical conditions, the hardness was measured at 295.3 HV1 with an AFR of 600 g/min, which is lower than the 300 g/min threshold. Figure 7d shows that the microhardness value of 312.5 HV1, obtained at 3000 bar pressure, an AFR of 300 g/min, and a traverse speed of 200 mm/min, is the highest among all the experimental combinations. This value demonstrates that the kinetic energy of the abrasive particles is maximized at high pressure, as is their ability to deform the surface. A significant increase in hardness is observed when the deformation hardening of TWIP980 steel, which has a high Mn content, is combined with surface strain hardening. Using an AFR of 600 g/min, the highest hardness value of 296.5 HV1 was obtained at a speed of 400 mm/min. However, the hardness value decreased to 294.3 HV1 at 200 mm/min with the same AFR. In general, it can be seen that pressure is the most effective parameter on microhardness. In terms of traverse speed, 200 mm/min is considered the most suitable, providing sufficient deformation time while maintaining the focusing ability of the abrasive. The effect of AFR depends on pressure and speed; 300 g/min provides consistent high hardness in most combinations. The 600 g/min level was only partially effective in some cases.

**Figure 8:**
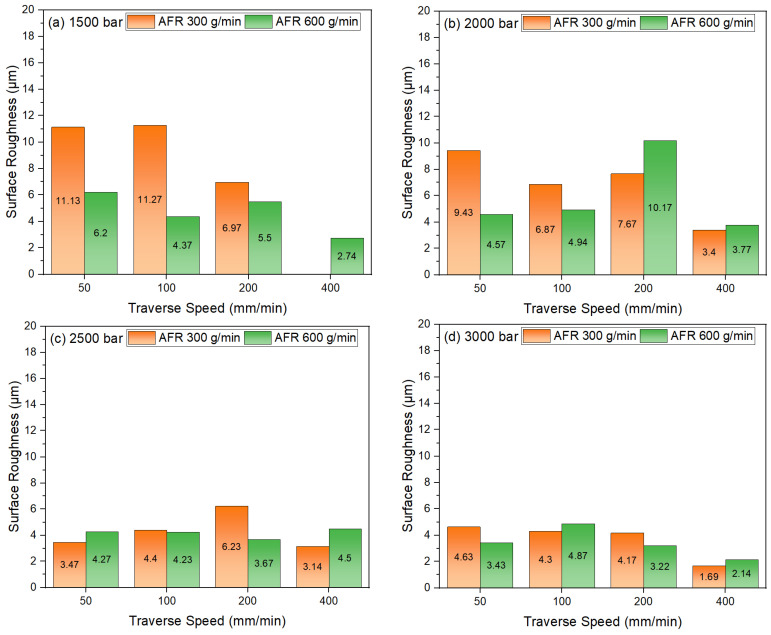
Traverse speed, pressure, and surface roughness relation: (**a**) 1500 bar; (**b**) 2000 bar; (**c**) 2500 bar; (**d**) 3000 bar.

Figure 8 shows the relationship between surface roughness (Ra) of TWIP980 steel sheet specimens cut under different water jet pressures, traverse speeds, and two different abrasive flows. High pressure, moderate traverse speed, and optimal AFR level are required to minimize surface roughness [37]. Figure 8a shows that quite high surface roughness values are obtained at low speeds for AFR 300 g/min when the surface roughness values obtained under 1500 bar pressure are analyzed. In particular, values of 11.13 µm and 11.27 µm were obtained at traverse speeds of 50 mm/min and 100 mm/min, respectively. These values suggest that irregular abrasion occurs on the surface due to insufficient abrasive kinetic energy at low pressure and AFR. At 200 mm/min, the Ra value decreases to 6.97 µm. However, no surface roughness data is available since cutting could not be performed at 400 mm/min. More regular and lower roughness values were obtained at an AFR of 600 g/min. Ra values of 6.20 µm at 50 mm/min, 4.37 µm at 100 mm/min and 5.50 µm at 200 mm/min were measured, as well as 2.74 µm at 400 mm/min. The higher abrasive rate is understood to provide more homogeneous abrasion of the material surface by increasing the number of particles carried. Figure 8b shows the Ra values measured for AFR at 300 g/min under a pressure level of 2000 bar: 9.43 µm at 50 mm/min, 6.87 µm at 100 mm/min, 7.67 µm at 200 mm/min, and 3.40 µm at 400 mm/min. These values indicate that surface irregularities decreased as the kinetic energy of the abrasive increased with pressure, thus improving surface quality. Using AFR 600 g/min yielded acceptable surface quality values of 4.57 µm at 50 mm/min, 4.94 µm at 100 mm/min, and 3.77 µm at 400 mm/min. However, the Ra value increased to 10.17 µm at a traverse speed of 200 mm/min. This abnormal increase is attributed to the deterioration of water jet coherence under high AFR and medium traverse speed conditions, with microscopic pitting and fluctuations occurring as a result of the uneven distribution of abrasive particles and the formation of secondary wear zones on the surface. It is reasonable to hypothesize that this phenomenon is due to excessive abrasive loading, which likely exceeds the water jet’s ability to maintain a stable and focused stream, resulting in particle turbulence and irregular impacts. The localized surface damage intensifies, causing a significant deterioration in surface integrity and roughness. Figure 8c presents the surface roughness values obtained when using AFR 300 g/min under a pressure of 2500 bar. These values are measured as 3.47 µm at 50 mm/min, 4.40 µm at 100 mm/min, 6.23 µm at 200 mm/min, and 3.14 µm at 400 mm/min. These results demonstrate that an increase in pressure generally improves surface quality; however, an increase in roughness is observed at 200 mm/min. At an AFR of 600 g/min, surface roughness remained stable and low, with values of 4.27 µm at 50 mm/min, 4.23 µm at 100 mm/min, 3.67 µm at 200 mm/min, and 4.50 µm at 400 mm/min. This demonstrates that a waterjet with a high AFR provides more consistent and uniform surface abrasion. Figure 8d presents the surface roughness values under 3000 bar pressure, which reach minimum levels. The Ra values for an AFR of 300 g/min are 4.63 µm at 50 mm/min, 4.87 µm at 100 mm/min, 4.17 µm at 200 mm/min, and 3.22 µm at 400 mm/min. Using AFR 600 g/min results in roughness values of 3.43 µm at 50 mm/min, 4.33 µm at 100 mm/min, 3.32 µm at 200 mm/min, and 1.69 µm at 400 mm/min. The lowest surface roughness obtained in all machining parameter combinations is 1.69 µm at 3000 bar pressure and 400 mm/min. The increase in kinetic energy carried by the waterjet under high pressure is thought to increase particle velocity and efficiency, providing a cleaner, smoother cut. At the same time, the controlled movement of the abrasive particles provides a smooth cut. It can be seen that pressure is the most important factor affecting surface roughness. The kinetic energy of the waterjet increases with pressure [38]. It is thought that this produces a more controlled and focused effect on the surface. The AFR affects both the quantity of abrasive particles transported and kinetic energy distribution to the surface. At extremely high AFR levels, collisions between particles can result in surface irregularities. Conversely, traverse speed can reduce roughness under low pressure conditions, though this effect is reduced under high pressure. The combination of high pressure (3000 bar), high traverse speed (400 mm/min), and an optimal AFR level (600 g/min) was found to provide the highest surface quality when water jet machining TWIP980 steel sheet material, by minimizing surface roughness.

**Figure 9:**
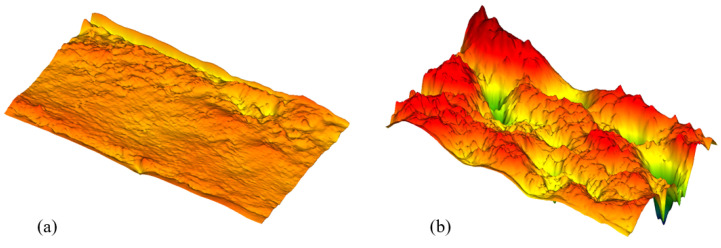
(**a**) Max surface quality 3000 bar, 400 mm/min traverse speed, 300 g/min AFR. (**b**) Min surface quality 1500 bar, 100 mm/min traverse speed, 300 g/min AFR.

Figure 9 compares the surface topographies of two TWIP980 steel sheet materials machined using the abrasive waterjet method in three dimensions. Figure 9a illustrates the machining conditions that produced the highest surface quality, while Figure 9b demonstrates the surface profile obtained under the lowest surface quality conditions. Figure 9a shows the surface topography of the sample that was cut using a water jet at 3000 bar pressure, with a traverse speed of 400 mm/min and an abrasive flow rate of 300 g/min. As can be seen in the image, the surface has a very homogeneous, continuous, and smooth structure. The high pressure enables the abrasive particles in the water jet to impact the surface at high speed, minimizing irregular material fracture [39,40]. At the same time, the high traverse speed limits contact time, resulting in a shallow, scarless cut mark. Meanwhile, the high AFR increases the density of particles reaching the surface, creating a more balanced, controlled micro-abrasion effect. This combination provides controlled energy distribution over the plastic deformation zone, thereby minimizing surface roughness. The smoothness of the topographic slope transitions and the low surface depth differences reflect this directly. Figure 9b, on the other hand, shows the 3D topographic profile of the surface processed under conditions of 1500 bar pressure, a traverse speed of 100 mm/min, and an AFR of 300 g/min. As can be seen in the image, the surface has a very wavy, hollow, and irregular structure. The low water jet pressure results in insufficient kinetic energy, preventing the abrasive particles from effectively removing material from the surface. A low traverse speed increases the contact time between the water jet and the surface, resulting in localized excessive wear, thermomechanical micro-wear, and non-directional surface deformation. Inadequate AFR reduces the number of particles the water jet carries, resulting in unevenly distributed microscopic cavities and wavy pits on the surface.

TWIP980 steel displays distinctive behavior during the process of abrasive water jet machining. This behavior is attributed to the steel’s fully austenitic microstructure and its high strain hardening capability. In contrast, TWIP steels demonstrate a tendency to absorb deformation more uniformly, resulting in smoother surface morphologies. These properties differentiate TWIP980 from advanced high-strength steels with regard to both machinability and surface integrity.

## 4. Conclusions

This study investigated the effects of basic process parameters, such as traverse speed, water jet pressure, and abrasive flow rate (AFR), on surface quality, geometrical accuracy, material removal rate, and microhardness in abrasive waterjet (AWJ) cutting of TWIP980 steel sheet material. The results obtained are given below.

- In terms of geometric accuracy criteria, such as kerf width and taper angle, the combination of high pressure and high traverse speed resulted in narrower and more symmetrical cutting geometries. In the range of 3000 bar, 400 mm/min, and 600 g/min, the kerf width was measured to be approximately 1.18 mm, and the taper angle was determined to be approximately 3.1°.
- The material removal rate (MRR) was optimized at combinations of high abrasive flow rate (AFR), traverse speed, and pressure. The maximum MRR value of 547 mm^3^/min was observed at 3000 bar, 400 mm/min, and 600 g/min parameters.
- The investigation revealed a positive correlation between deformation and hardening, particularly at higher pressures and moderate speeds. The highest microhardness value of 312.5 HV1 was obtained at 3000 bar, 200 mm/min, and 300 g/min. This finding indicates an increase in the local dislocation density on the material surface.
- The investigation showed that the water jet pressure was the most effective parameter in reducing surface roughness. Higher pressure levels are proven to increase the kinetic energy of the water jet, resulting in a more controlled and uniform abrasive effect on the surface and lower Ra values. The lowest surface roughness value of 1.69 µm was obtained at the combination of 3000 bar, 400 mm/min traverse speed, and 600 g/min AFR.
- The 3D surface topography analyses demonstrated that cutting parameters directly influence surface morphology under optimal and suboptimal surface roughness conditions. Higher energy densities and abrasive particle fluxes are shown to produce more homogeneous and oriented surface geometries, while low energy conditions produce irregular, indented, and rough surfaces.
- At a pressure of 1500 bar, a traverse speed of 400 mm/min, and an abrasive flow rate (AFR) of 300 g/min, AWJ cutting was not possible.

## Figures and Tables

**Table 1:** Chemical composition of TWIP980 steel (wt.%).

Steel	C	Mn	Si	P	S	Al	Fe
TWIP980	0.64	16.69	0.042	0.012	0.001	1.66	Bal.

**Table 2:** Input process parameters and levels.

Parameters	Factor Levels			
	1	2	3	4
Traverse Speed (mm/min)	50	100	200	400
Pressure (bar)	1500	2000	2500	3000
Abrasive Flow Rate (g/min)	300	600		

## Data Availability

The original contributions presented in this study are included in the article. Further inquiries can be directed to the corresponding author.
